# Relationship Between Hepcidin, Iron Metabolism, Inflammation and Hypersplenism in Anaemia of Kala‐Azar

**DOI:** 10.1111/pim.70014

**Published:** 2025-07-10

**Authors:** Alyne Ferreira De Almendra Freitas, Adelino Soares Lima Neto, Camila Maria Coelho de Moura, Giovana Dias Silva, Marília de Sousa Araújo Barbosa e Silva, Keline Medeiros de Araújo Vilges, Francisco Mateus Alves de Morais Ferreira, Dorcas Lamounier Costa, Carlos Henrique Nery Costa

**Affiliations:** ^1^ Postgraduate Program in Health and Science Federal University of Piauí Teresina Piauí Brazil; ^2^ Leishmaniasis Research Laboratory – LabLeish Piauí Brazil; ^3^ Dr. Costa Alvarenga Central Public Health Laboratory (LACEN) Teresina Piauí Brazil; ^4^ Federal University of Piauí Teresina Piauí Brazil; ^5^ Center for Intelligence on Emerging and Neglected Tropical Diseases (CIATEN) Teresina Piauí Brazil; ^6^ Federal University of Paraíba João Pessoa Paraíba Brazil; ^7^ University of Brasilia Campus Darcy Ribeiro Brasilia ‐ DF Brazil; ^8^ Municipal Government of João Pessoa‐PB João Pessoa Paraíba Brazil

**Keywords:** anaemia, hepcidin, hypersplenism, inflammation, iron metabolism, kala‐azar, visceral leishmaniasis

## Abstract

Kala‐azar, or visceral leishmaniasis (VL), is a parasitic disease caused by *Leishmania* spp., characterised by fever, weight loss, splenomegaly, hepatomegaly, and anaemia. This study evaluated the relationship between hepcidin, inflammation, iron metabolism, and hypersplenism in VL‐associated anaemia. In this cross‐sectional study, confirmed VL patients without recent transfusions were assessed. Haematological and inflammatory parameters were analysed using correlation and multivariate regression tests. Anaemia was present in 95.2% of the sample, predominantly normocytic (59.5%) and normochromic (76.2%), or microcytic (40.5%) and hypochromic (23.8%). Inflammatory markers were markedly elevated in most patients, particularly hepcidin, which was increased in 97.6% of cases (median: 351.46 ng/mL), suggesting persistent inflammation and impaired iron bioavailability. However, IL‐6, CRP, and ferritin showed weak to moderate negative correlations with hepcidin (*ρ* = −0.33, *ρ* = −0.66, and *ρ* = −0.30, respectively). These findings highlight the complex interplay between anaemia and inflammation in kala‐azar, with elevated hepcidin levels and paradoxical correlations with inflammatory markers. They underscore the central role of splenomegaly in VL‐related anaemia and suggest potential contributions from other factors affecting iron metabolism, such as erythropoietin and erythroferrone. Understanding the dynamics of these markers throughout disease progression and treatment may further elucidate the pathophysiology of VL and support the development of targeted therapies.

## 1 Introduction

Kala‐azar, also known as visceral leishmaniasis (VL), is a severe infectious disease caused by protozoa of the genus *Leishmania*, primarily transmitted through the bites of infected *Phlebotomus* sandflies [1, 2, 3, 4]. Globally, VL remains a significant public health issue, particularly in tropical and subtropical regions [5]. The disease is endemic in several developing countries where socio‐economic challenges and limited healthcare infrastructure facilitate its spread [5, 6]. Clinically, VL is characterised by prolonged fever, large splenomegaly, hepatomegaly, anaemia, and weight loss, often leading to high morbidity and mortality if left untreated [7, 8, 9, 10].

Anaemia is a common complication in kala‐azar [1, 8]. Its aetiology is multifactorial, involving a complex interplay between infection, hypersplenism, immune response, inflammation, and disruptions in iron metabolism [4, 8, 11]. Chronic inflammation induced by *Leishmania* infection stimulates the production of pro‐inflammatory cytokines such as interleukin‐6 (IL‐6) and tumour necrosis factor‐alpha (TNF‐α), which play critical roles in regulating hepcidin, a key iron‐regulatory hormone [12, 13]. Elevated hepcidin levels promote iron sequestration in macrophages, inhibit intestinal iron absorption, and decrease iron release from storage, ultimately contributing to anaemia by limiting iron availability for erythropoiesis [14, 15, 16]. Therefore, chronic disease anaemia, commonly observed in persistent infections like VL, is characterised by altered iron metabolism driven by inflammatory responses. This type of anaemia reflects disease severity and influences patient prognosis and treatment outcomes [17, 18]. Understanding the underlying mechanisms of anaemia in VL, particularly the roles of inflammatory mediators and iron‐regulatory proteins, is essential for developing effective therapeutic strategies.

Hepcidin is considered the primary homeostatic regulator of iron metabolism. Its expression is predominantly regulated by feedback mechanisms dependent on circulating iron status. Additionally, its synthesis is influenced by inflammatory processes and erythropoietic activity. Iron‐mediated signalling involves distinct molecular pathways that enable hepatocytes to sense systemic iron levels [19].

This sensing can occur indirectly, in response to the production of bone morphogenetic proteins (BMPs), which are induced by iron in hepatic sinusoidal endothelial cells. These proteins activate BMP receptors, leading to phosphorylation and SMAD signalling, which translocates to the nucleus and results in the activation of hepcidin gene transcription [19, 20].

Furthermore, hepatocytes directly sense iron through the expression of transferrin receptors (TfR1, TfR2) and the hemochromatosis protein HFE. Under conditions of iron overload, increased expression of TfR2 and formation of the HFE/TfR2 complex, in interaction with hemojuvelin (HJV), a BMP‐specific co‐receptor, amplify BMP pathway signalling and enhance hepcidin expression [15, 19, 20, 21].

During inflammation, hepcidin is primarily induced by interleukin‐6 (IL‐6), which activates the STAT3 signalling pathway in hepatocytes, leading to hepcidin transcription. Moreover, IL‐6 may exert a secondary suppressive effect on erythroid precursors, and inhibition of BMP signalling compromises IL‐6‐induced hepcidin expression [19, 20].

Despite advancements in kala‐azar treatment, managing anaemia remains challenging due to its multifactorial nature. Several factors, including nutritional deficiencies, splenic sequestration of red blood cells, and inflammatory activity compound the complexity of anaemia in VL [4, 7]. Additionally, the potential involvement of less‐studied regulators such as erythroferrone and erythropoietin (EPO) further complicates the pathophysiological landscape [15, 16].

Erythroferrone is a hormone produced by erythroblasts in response to erythropoietin, which promotes the inhibition of hepcidin synthesis by hepatocytes through the suppression of the bone morphogenetic protein (BMP) signalling pathway, resulting in increased iron availability for haemoglobin synthesis [19, 20].

The present study investigates the relationship between inflammatory activity and iron metabolism in VL patients, focusing on key markers such as hepcidin, ferritin, IL‐6, and C‐reactive protein (CRP). By exploring these correlations, this research seeks to deepen the understanding of VL‐associated anaemia's pathophysiology and identify potential therapeutic targets. The findings may contribute to improved clinical management and the development of novel interventions to mitigate anaemia and enhance the quality of life for patients affected by visceral leishmaniasis.

## 2 Methods

The study was conducted in Teresina, the capital of Piauí, a state in northeastern Brazil recognised as an important healthcare reference center for the Northern and Northeastern regions of the country. The research was conducted at the Natan Portela Institute of Tropical Diseases (Instituto de Doenças Tropicais Natan Portela—IDTNP) a regional hospital specialised in infectious and parasitic diseases. This institute collaborates closely with the Federal University of Piauí (Universidade Federal do Piauí—UFPI). The study obtained approval from the Research Ethics Committee of UFPI through the Plataforma Brasil system (CAAE: 49960215.3.0000.5214), as confirmed by the committee's substantiated opinion, documented as number 1.544.071. In this study, we focused on patients diagnosed with visceral leishmaniasis, representing individuals of all genders and various age groups. Those who had received blood transfusions were excluded from the research. A confirmed case was defined as a patient displaying key clinical symptoms, including fever, splenomegaly, pallor, and weight loss, supported by laboratory tests that established the etiological diagnosis.

### 2.1 Data Collection

The laboratory tests employed to confirm visceral leishmaniasis included a rapid diagnostic test for kala‐azar and the direct detection of *Leishmania* in bone marrow. Evidence of the disease was established by visualising amastigote forms in bone marrow smears or growth in the culture of a bone marrow aspirate.

Blood count was obtained through routine kala‐azar examinations at IDTNP. Anaemia was characterised by haemoglobin levels below established normal thresholds, according to WHO criteria: less than 13 g/dL for men, less than 12 g/dL for women, and less than 11 g/dL for pregnant women and children aged 6 months to 5 years.

C‐reactive protein (CRP) levels and serum iron levels and related parameters, including ferritin, total iron‐binding capacity, and transferrin saturation, were measured. The levels of IL‐6 in all study participants were determined at Lableish, IDTNP, using the BD Cytometric Bead Array (CBA) Human IL‐6 Enhanced Sensitivity Flex Set Kit on a FACS CANTO 2 flow Cytometer (Becton Dickinson, New Jersey, USA), and concentration values were recorded for most participants (34 patients). For this purpose, a 25 μL aliquot of serum was centrifuged, and serum or plasma aliquots were preserved for subsequent assays.

This study assessed hepcidin levels in 42 patients using the ELISA method with the Human Hepcidin ELISA Kit (abx051456—Abbexa Ltd., Cambridge Science Park, Cambridge, CB4DEY, UK). For the assay's detection range, samples were diluted at 1:20 ratio.

### 2.2 Statistical Analysis

The study utilised 42 samples and analysed 30 variables. Key variables include sex, age, hepcidin, interleukin‐6, C‐reactive protein, erythrocytes, haemoglobin, haematocrit, serum iron, transferrin saturation index, ferritin, total iron‐binding capacity, and others related to iron metabolism and haematological parameters. The statistical analysis employed included boxplots to evaluate data dispersion, medians, and outliers, Spearman's and Pearson's correlation to assess relationships between variables, and multivariate linear regression to determine the combined influence of variables on outcomes. Graphs and statistical calculations were performed using OriginPro 2021 9.8.0.200.

## 3 Results

The sample was predominantly composed of men, with 83.3% of participants identifying as male and 16.7% female, highlighting a significant gender imbalance in the study population. Regarding age distribution, the study primarily included individuals aged 16 to 40, indicating that it most represented this age group. All of the patients studied had splenomegaly at diagnosis. HIV serology was knew in 33 of the 42 patients studied and co‐infection was identified in eight of them (24.0%). None of the patients died during the hospital stay.

**FIGURE 1:**
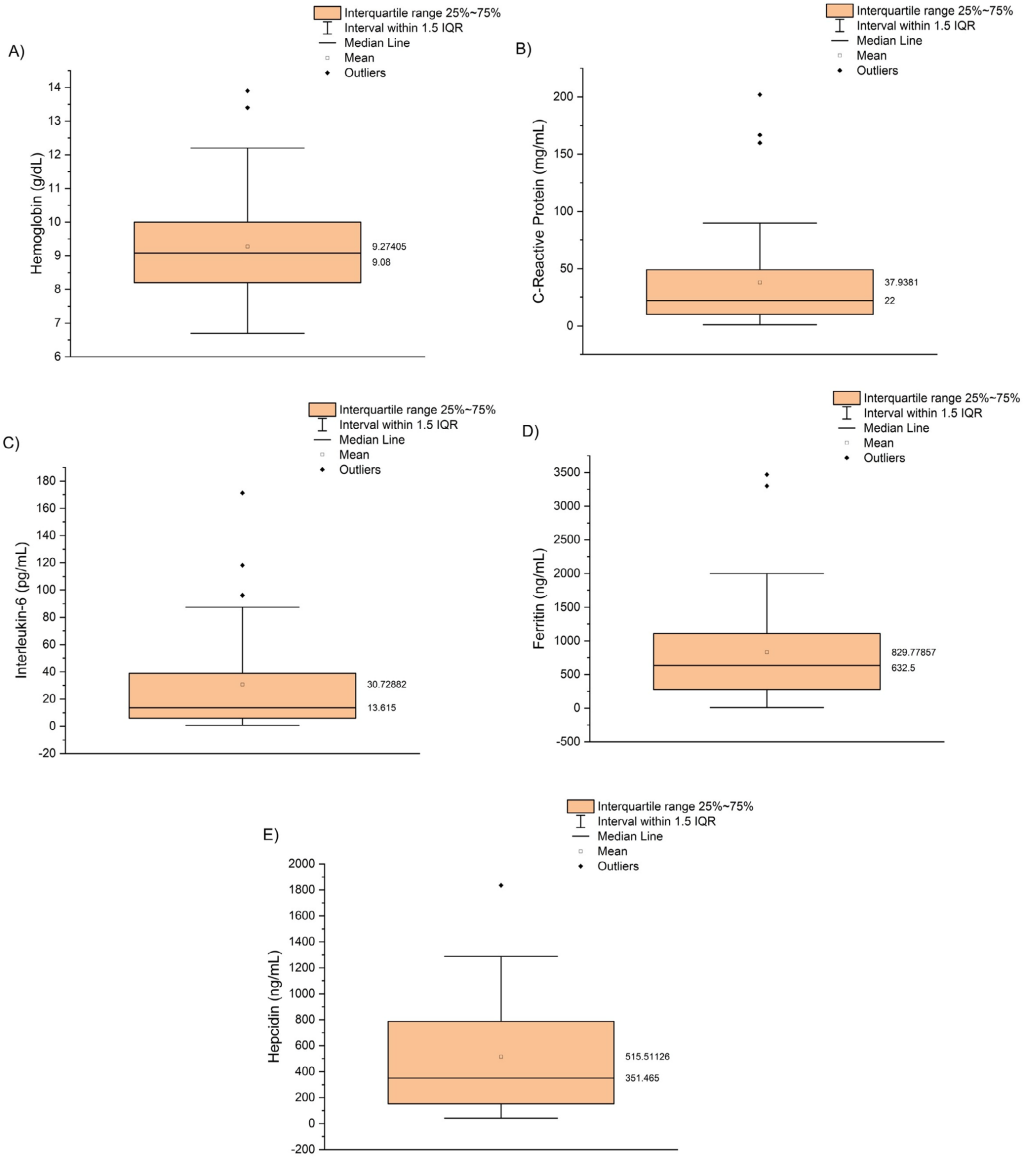
Boxplots showing the distribution of key markers in patients with visceral leishmaniasis. (A) Haemoglobin, (B) C‐reactive protein (CRP), (C) interleukin‐6 (IL‐6), (D) ferritin, and (E) hepcidin.

Regarding haemoglobin, the mean value recorded was 9.3 g/dL (Figure 1A; Table 1), indicating a low average haemoglobin concentration in the blood. According to WHO criteria, 95.2% of the patients studied had anaemia.

**TABLE 1:** Descriptive statistics of anaemia indicators and body iron.

	Haemoglobin	VCM*	HCM**	Serum iron	Transf. sat. index
Mean	9.27	80.56	26.60	59.51	22.70
SD	1.66	6.55	2.78	28.86	12.73
CI	8.75; 9.79	78.5; 82.6	25.7; 27.5	50.5;68.5	18.7; 26.7

* Mean corpuscular volume.

** Mean corpuscular haemoglobin.

The morphological classification of anaemia was conducted using mean corpuscular volume (MCV) and mean corpuscular haemoglobin concentration (MCHC). Anaemia was classified based on MCV as microcytic, normocytic, or macrocytic. Microcytic anaemia was defined as MCV < 80 fL, normocytic anaemia as MCV between 80 and 100 fL, and macrocytic anaemia as MCV > 100 fL. In addition, MCHC was used to further classify anaemia as hypochromic or normochromic. Hypochromic anaemia was defined as MCHC < 32 g/dL, while normochromic anaemia was defined as MCHC ≥ 32 g/dL.

The results of the classification, presented in Table 2, revealed that 17 patients were identified as having microcytic anaemia, while 25 patients were classified as normocytic. Regarding the MCHC‐based classification, 10 patients presented with hypochromic anaemia, whereas 32 patients were categorised as normochromic. These findings highlight the predominance of normocytic anaemia (59.5%) within the study population based on MCV, with an impressive proportion of microcytic anaemia cases (40.5%). Regarding haemoglobin concentration in erythrocytes, normochromic anaemia predominated (76.2%), with fewer cases classified as hypochromic anaemia (23.8%).

**TABLE 2:** Morphological classification of anaemia based on mean corpuscular volume (MCV) and mean corpuscular haemoglobin concentration (MCHC).

Type of anaemia	Classification criteria	Number of patients (*n*)	Percentage (%)
Microcytic	MCV < 80 fL	17	40.5
Normocytic	80 fL ≤ MCV ≤ 100 fL	25	59.5
Macrocytic	MCV > 100 fL	0	0
Hypochromic	MCHC < 32 g/dL	10	23.8
Normochromic	MCHC ≥ 32 g/dL	32	76.2

*Note:* The table shows the number and percentage of patients in each category.

To assess body iron, serum iron and transferrin saturation were analysed. Regarding serum iron, the mean value found was 59.5 μg/dL and the median was 55 μg/dL (Table 1). The transfer saturation index, which indicates the proportion of transfer saturated with iron, showed a mean of 22.7% (Table 1).

In addition, ferritin and hepcidin were examined to assess iron metabolism. Ferritin, listed as an iron storage indicator but also an acute phase protein of inflammation, showed a mean value of 829.8 ng/mL and the median was 632.5 ng/mL (Figure 1D; Table 3). Furthermore, the standard deviation of 792.3 ng/mL indicates considerable variability in iron stores among participants, with a minimum value of 8.6 ng/mL. Approximately 74% of the sample had elevated ferritin levels.

**TABLE 3:** Descriptive statistics of inflammatory key markers.

	CRP*	Il‐6	Ferritin	Hepcidin
Mean	37.94	30.73	829.78	515.51
SD	44.66	38.72	792.29	432.57
CI	26.44; 53.44	19.02; 42.44	589.19; 107.37	384.79; 646.23

* C‐reactive protein.

Similarly, hepcidin, another key marker of iron metabolism, had a mean value of 515.5 ng/mL and the median was 351.4 ng/mL (Figure 1E, Table 3). The standard deviation of 432.6 ng/mL (Table 3). Only one patient had normal hepcidin levels, while all the others exhibited elevated (17%) or extremely high levels (83%), exceeding 100 ng/mL.

CRP, one inflammatory marker, showed a mean value of 37.9 mg/dL and the median was 22.0 mg/dL (Figure 1B; Table 3). All patients had elevated CRP levels.

Regarding interleukin‐6 (IL‐6), a pro‐inflammatory cytokine, the mean value recorded was 30.7 pg/mL and the median was 16.61 pg/mL (Figure 1C; Table 3). Approximately 70% of the patients had elevated IL‐6 levels.

**FIGURE 2:**
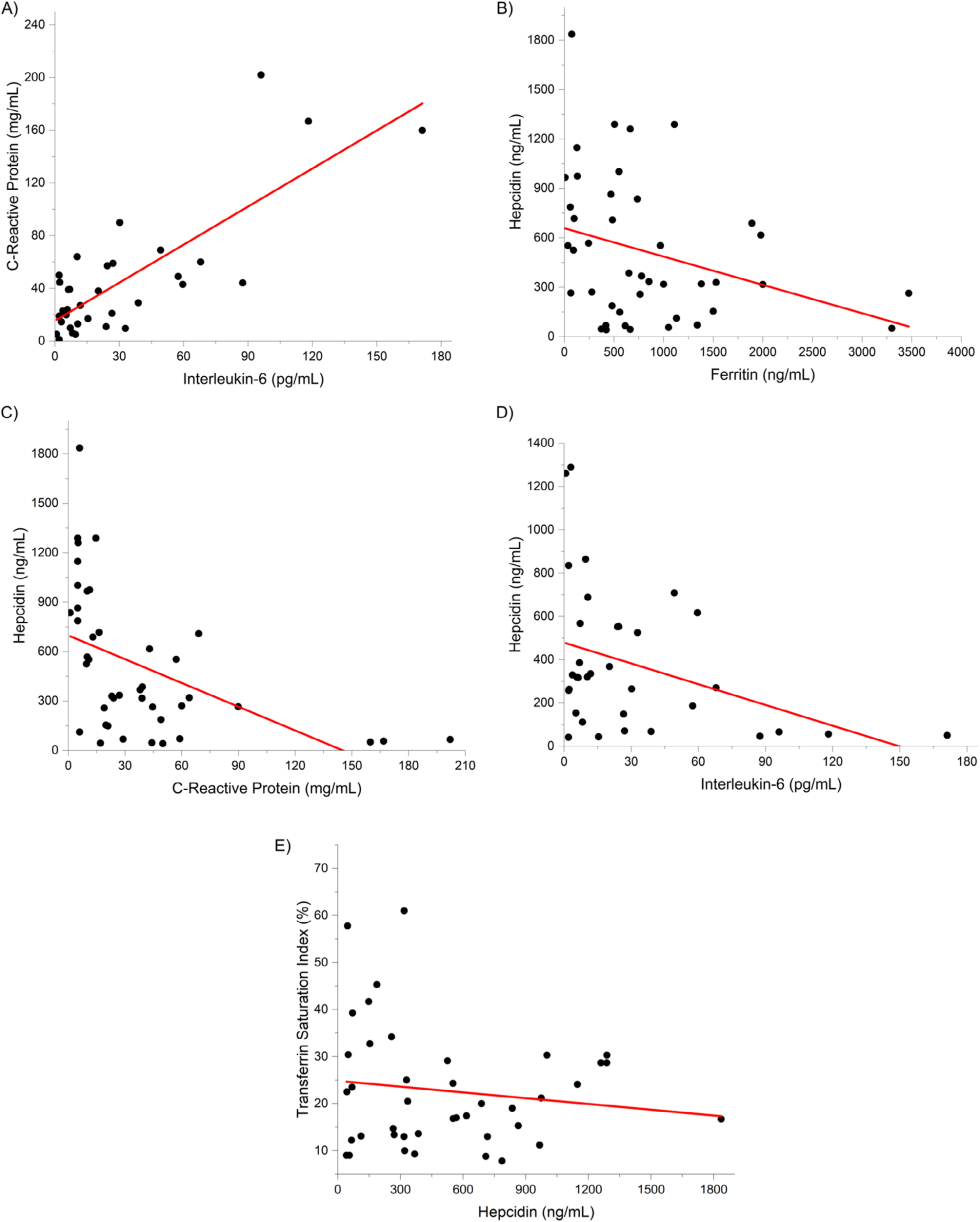
Scatter plots showing significant associations between inflammatory and iron metabolism markers: (A) CRP versus IL‐6, (B) Hepcidin versus Ferritin, (C) Hepcidin versus CRP, (D) Hepcidin versus IL‐6, and (E) Transferrin saturation index versus Hepcidin.

Figure 2A illustrates a strong positive correlation between interleukin‐6 (IL‐6) and C‐reactive protein (CRP), with a Pearson correlation coefficient (𝑟 = 0.79). Higher IL‐6 levels were strongly associated with increased CRP levels, reflecting the role of IL‐6 in driving systemic inflammation. The fitted line indicates that for every unit increase in IL‐6, CRP rises by approximately 0.96 mg/mL. The *R* ^2^ value of 0.63 suggests that about 63% of the variation in CRP was explained by IL‐6 levels, with the F‐test statistically significant (𝑝 < 0.001).

Figure 2B explores the relationship between ferritin and hepcidin, revealing a moderate negative correlation (𝑟 = −0.31). This suggests that higher ferritin levels are associated with lower hepcidin levels. The regression analysis shows that hepcidin decreases by approximately 0.17 ng/mL for each unit increase in ferritin. However, the *R* ^2^ value 0.09 indicates a weak relationship, suggesting other factors may significantly influence hepcidin levels. The association is statistically significant (𝑝 = 0.04).

**FIGURE 3:**
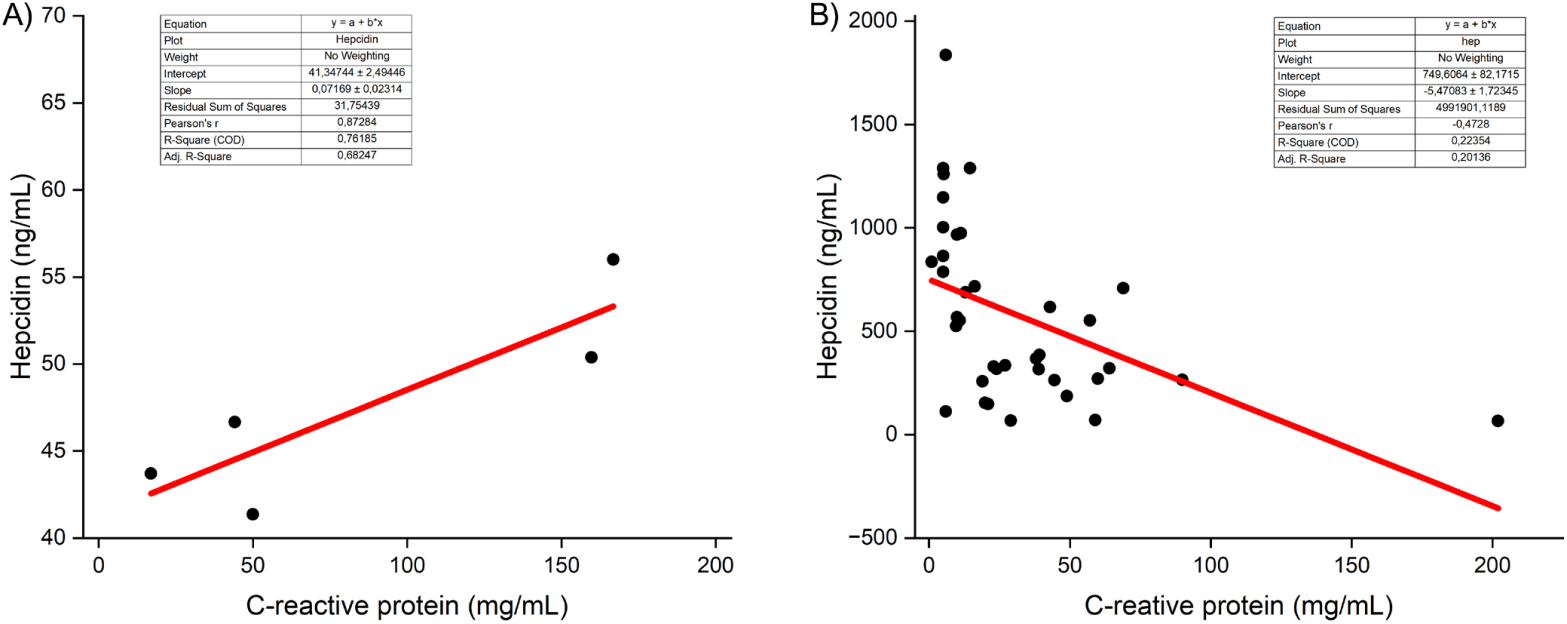
Scatter plots showing significant associations between hepcidin and CRP (A) CRP versus hepcidin < 60 ng/mL (B) CRP versus hepcidin > 60 ng/mL.

Figure 2C highlights a moderate negative correlation between CRP and hepcidin (𝑟 = −0.50). The fitted line shows a decrease of approximately 4.80 ng/mL in hepcidin for every unit increase in CRP. The *R* ^2^ value of 0.25 indicates that about 25% of the variation in hepcidin can be explained by CRP levels, demonstrating a stronger relationship compared to ferritin. This association is highly significant (𝑝 < 0.001). Due to this unexpected result, a segmental analysis of the relationship between hepcidin and CRP was conducted (Figure 3). The relationship was assessed in five patients with lower hepcidin levels, slightly above the normal value of 50 ng/mL, but below 60 ng/mL (Figure 3A) and in 37 patients above this level (Figure 3B). The correlation remained strongly negative in patients with higher hepcidin concentration (𝑟 = −0.47; 𝑝 = 0.003). On the other hand, the correlation between hepcidin and CRP reversed, becoming positive in the patients with lower hepcidin levels (𝑟 = 0.87; 𝑝 = 0.053).

Figure 2D examines the correlation between interleukin‐6 (IL‐6) and hepcidin, showing a moderate negative association (𝑟 = −0.38). Higher IL‐6 levels are associated with a reduction in hepcidin. The fitted line indicates a decrease of approximately 3.19 ng/mL in hepcidin for each unit increase in IL‐6. The *R* ^2^ value of 0.14 suggests that IL‐6 levels account for 14% of the variability in hepcidin, with the association being statistically significant (𝑝 = 0.028).

Figure 2E examines the relationship between transferrin saturation index (TSI) and hepcidin, showing a weak negative correlation (𝑟 = −0.14). The fitted line indicates that the TSI decreases by approximately 0.004% for every unit increase in hepcidin. The *R* ^2^ value of 0.02 suggests that only about 2% of the variation in TSI can be explained by hepcidin levels, indicating a very weak association. This relationship is not statistically significant (𝑝 = 0.382).

**FIGURE 4:**
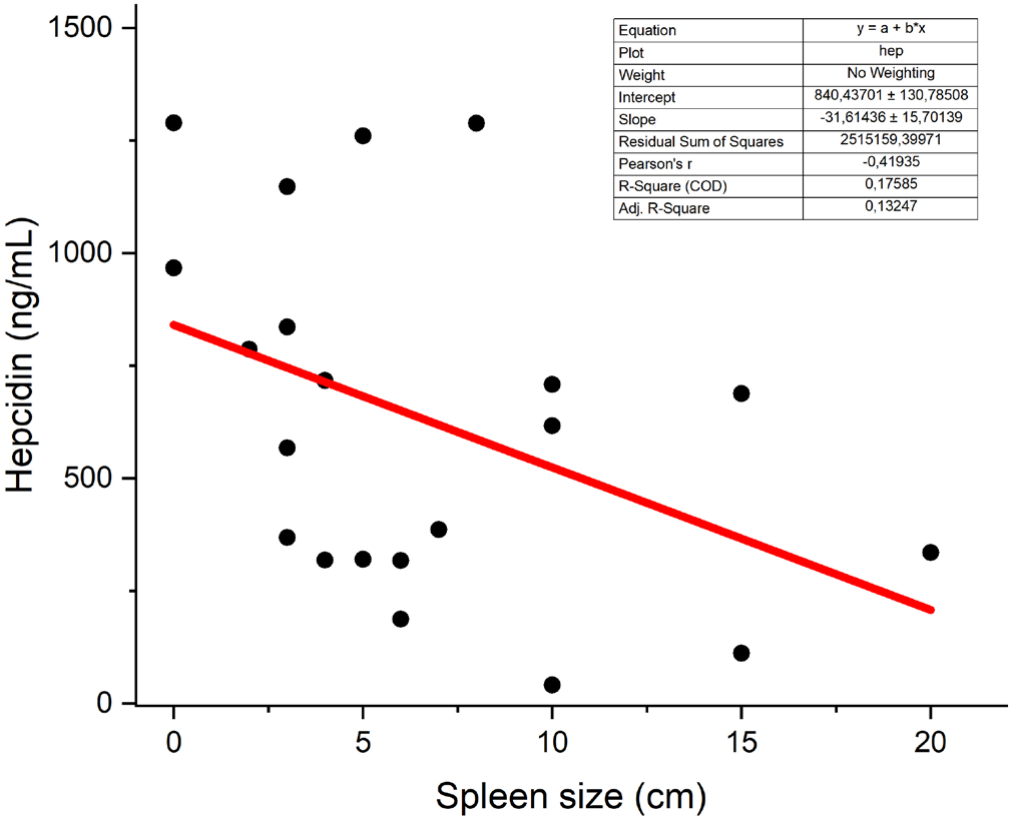
Scatter plots showing significant associations between spleen size and hepcidin.

Additionally, the correlation between hepcidin and spleen size was analysed, revealing a negative Pearson's r (𝑟 = −0.48) with statistical significance (𝑝 = 0.03) (Figure 4).

Multivariate linear regression analysis was performed to explore the relationship between haemoglobin levels and selected predictors, including inflammatory markers, iron indicators, and other factors. Table 4 summarises the results, with haemoglobin as the dependent variable and serum iron, transferrin saturation index, total iron‐binding capacity (TIBC), ferritin, and hepcidin as independent variables. The model explained 22.6% of the variation in haemoglobin levels (𝑅^2^ = 0.226).

**TABLE 4:** Multivariate linear regression results for haemoglobin as the dependent variable.

Independent variables	Coefficient (*β*)	Std. Error	*t*	*p*	95% CI
Constant	8.5610	1.617	5.294	0.000**	[5.278, 11.844]
Serum iron	−0.0136	0.023	−0.594	0.556	[−0.060, 0.033]
Transferrin saturation index	0.0340	0.053	0.647	0.522	[−0.073, 0.141]
Total iron‐binding capacity	0.0020	0.006	0.360	0.721	[−0.009, 0.013]
Ferritin	−0.0006	0.000	−1.534	0.134	[−0.001, 0.000]
Hepcidin	0.0013	0.001	1.976	0.056*	[−3.45e‐05, 0.003]

*Note:* *R* ^2^ = 0.226; Adjusted *R* ^2^ = 0.115; Condition Number = 7.33e+03.

* Indicates marginal significance (*p* < 0.10).

** Indicates strong statistical significance (*p* < 0.05).

Among the predictors, hepcidin was the only variable showing statistical significance (𝑝 = 0.056), with a positive coefficient (0.0013), indicating a slight association between higher hepcidin levels and increased haemoglobin. Other predictors, such as serum iron (−0.0136), transferrin saturation (0.0340), TIBC (0.0020), and ferritin (−0.0006), showed no statistically significant impact (𝑝 > 0.05). Additionally, the high Condition Number highlights potential multicollinearity, suggesting that some predictors may be highly correlated, reducing the reliability of the individual coefficients.

## 4 Discussion

This study analysed the association between alterations iron metabolism and activity inflammatory in patients diagnosed with VL, contributing to a better understanding of the interaction between anaemia and inflammation in the disease's pathology.

The collected data indicate that the majority of the evaluated patients are male. The predominance of visceral leishmaniasis in male individuals has been associated with the action of testosterone, which can influence the immune response to *Leishmania* infection [21]. Studies suggest that this male hormone modulates innate, humoral, and cell‐mediated immune responses, making men more susceptible to developing the symptomatic form of the disease. This factor, combined with behavioural and environmental differences, helps explain the higher incidence and severity of visceral leishmaniasis in men [4, 5, 18, 21]. The predominance of patients aged 16 to 40 years, along with the small sample of child individuals, indicates that we had more access to the young adult population during the study period.

The results demonstrated that anaemia was present in most patients, which is consistent with the literature identifying it as a common symptom of VL [7, 8]. The analysis revealed that patients exhibited normocytic and normochromic anaemia, characteristic of chronic inflammation, or microcytic and hypochromic anaemia, typical of iron deficiency due to altered iron availability for haemoglobin production [7, 22, 23]. Previous studies, have shown that anaemia normalisation occurs in nearly all kala‐azar patients after treatment, even without iron supplementation, arguing against primary iron deficiency and in favour of altered iron metabolism [7]. This type of anaemia is frequently associated with persistent inflammation and increased hepcidin levels, a protein that regulates iron homeostasis by limiting iron availability to the bone marrow and other tissues [14, 15, 16, 23].

Searches have examined the pathophysiological processes of iron deficiency in inflammatory conditions, showing that it is triggered by pro‐inflammatory cytokines (IL‐6, IL‐1, TNF‐α) [22, 24]. The signalling pathways activated by these cytokines lead to blunted responses to erythropoietin (EPO), apoptosis of erythroid progenitors, impaired absorption mediated by hepcidin, and iron sequestration by the reticuloendothelial system (RES) [25, 26, 27].

Additionally, the analysis of inflammatory markers demonstrated universally elevated CRP values and increased IL‐6 in most patients. They are associated with greater inflammation severity, reinforcing the central role of the inflammatory response in VL pathogenesis, triggered by the innate immune response via IL‐6 [13, 24, 28, 29]. In this context, IL‐6 binds to hepatocyte membrane receptors and induces the acute‐phase response, leading to increased secretion of proteins such as CRP, hepcidin and ferritin [13, 25, 28]. The strong positive linear correlation between IL‐6 and CRP highlights the intense inflammatory state inherent to kala‐azar [24, 28, 30].

The assessment of iron profiles revealed that most patients had low iron levels, normal or reduced transferrin saturation (TSI) and predominantly elevated ferritin, indicating insufficient iron availability for haemoglobin production. Serum ferritin and transferrin saturation are the most critical parameters for defining iron deficiency [22, 23]. It is worth noting that both TSI and, particularly, ferritin are valid measures; however, both can vary due to inflammatory conditions. Studies have shown that TSI is less affected by inflammatory processes and may therefore be more accurate and reliable than serum ferritin, especially in conditions with heightened inflammation [22, 23].

Moreover, the high absolute levels of hepcidin and ferritin indicate the involvement of inflammatory mechanisms that regulate iron metabolism. The interaction between anaemia and inflammation is well established, with alterations in iron metabolism primarily driven by increased hepcidin production [9, 28, 29, 31, 32, 33]. During the acute‐phase response, hepcidin levels rise, resulting in limited intestinal iron absorption and inhibition of iron release from stores [14, 16, 20]. This increase in hepcidin is a critical factor in regulating ferritin levels and modulating the inflammatory response, leading to iron deficiency anaemia even in patients with normal iron stores [15, 24, 32].

Hepcidin functions as an adaptive defence mechanism aimed at limiting iron availability to pathogens, as many microorganisms require iron for survival and replication [26, 32, 33]. Increased hepcidin levels induce the ubiquitination, internalisation, and inactivation of ferroportin, a protein responsible for iron absorption from the intestinal lumen into enterocytes and iron export from macrophages into circulation, ultimately reducing iron availability for erythropoiesis [30, 31, 32].

However, despite the elevated levels of inflammatory markers such as CRP, IL‐6, and ferritin, as well as the significant increase in hepcidin, the correlations between hepcidin and these other markers were negative. This is unexpected, since hepcidin, like ferritin and CRP, is an acute‐phase protein typically secreted in response to IL‐6 stimulation, which would usually result in a positive correlation [34, 35, 36, 37, 38].

The segmental assessment of the relationship between hepcidin and CRP demonstrated that this trend was reversed in the few patients who had normal or near‐normal hepcidin levels. These findings suggest that at physiological levels, hepcidin behaves as expected based on existing literature [7, 9, 10, 37], whereas at elevated concentrations, it seems to function via a negative feedback mechanism. Further exploration of other coexisting factors involved in the regulation of iron metabolism and the development of anaemia in kala‐azar could provide valuable insights.

Splenomegaly, a hallmark of VL, plays a critical role in anaemia pathogenesis [1, 4, 7, 35, 36, 37]. Spleen enlargement and subsequent reticuloendothelial hyperplasia increase blood cell sequestration, contributing to anaemia onset and further exacerbating the inflammatory process through macrophage activation, which, in turn, shortens the lifespan of red blood cells [7, 10, 25, 26, 35]. The data revealed a significant positive correlation between splenomegaly and the inflammatory markers such as CRP, IL‐6 and ferritin, reinforcing the strong link between splenic involvement and inflammation. Conversely, the negative interactions observed between splenomegaly and both anaemia and hepcidin suggest the influence of additional regulatory factors that modulate hepcidin activity in response to tissue hypoxia, ultimately impacting iron metabolism [26, 35].

Initially, the inflammation and hepcidin levels rise, contributing to anaemia development. However, as anaemia progresses, tissue hypoxia stimulates erythropoietin (EPO) and erythroferrone production—both known to suppress hepcidin [15, 16, 38, 39].

EPO is produced by the kidneys in response to hypoxia and stimulates the bone marrow to produce red blood cells [15, 16, 25, 33]. However, in cases of chronic inflammation such as VL, EPO production may be insufficient due to inflammatory cytokines (such as IL‐6), which inhibit the erythropoietic response [27, 32, 33]. Chronic inflammation not only reduces the production of EPO, but can also generate resistance to its action, which compromises the bone marrow's ability to produce red blood cells, even in the presence of normal levels of EPO, contributing to the existence of anaemia, regardless of the role of hepcidin [16, 32, 37, 38].

In turn, erythroferrone, produced during erythropoiesis, inhibits the release of stored iron for red blood cell production [15, 16, 33, 36, 39]. Under inflammatory conditions like VL, erythroferrone deficiency can block iron release, exacerbating anaemia and reducing the efficacy of iron supplementation [16, 36, 39].

Thus, understanding the role of hepcidin and the mechanisms regulating its expression during the exacerbated inflammatory state characteristic of visceral leishmaniasis may provide important contributions to the clinical management of iron metabolism disturbances associated with this disease, as well as guide the development of more effective therapeutic interventions. It is worth noting that inhibition of the BMP signalling pathway, which drives hepcidin transcription, impairs its IL‐6‐mediated induction and may therefore represent a potential therapeutic target for modulating this pathway in cases of anaemia of inflammation [20].

This study provided a comprehensive analysis of the relationship between inflammatory activity and alterations in iron metabolism in patients with visceral leishmaniasis (VL), highlighting the marked elevation of hepcidin and its negative correlation with other inflammatory markers, which underscores the complexity of anaemia associated with the disease. Splenomegaly, along with activation of the phagocytic and inflammatory systems, plays a central role in the dysregulation of iron metabolism. However, the mechanisms that control and regulate hepcidin expression remain incompletely understood.

Understanding inflammatory markers and their correlations with haematological and iron metabolism parameters is essential for elucidating the pathophysiology of VL and may support the development of more effective therapeutic strategies. Investigating the temporal dynamics of these markers, such as hepcidin, ferritin, erythropoietin (EPO), and erythroferrone, throughout the course of the disease and during treatment, as well as their relationship with splenomegaly, may provide valuable insights into the underlying mechanisms of VL‐associated anaemia and its association with clinical severity.

## Author Contributions

C.H.N.C. and D.L.C. contributed to the study's conception and design, performed data analysis and interpretation, provided analytical tools, supervised the research process, and critically reviewed and revised the manuscript. A.F.D.A.F. contributed to data analysis and interpretation and drafted the initial version of the manuscript. A.S.L.N., C.M.C.M., and F.M.A.M.F. conducted the laboratory procedures. G.D.S. was responsible for patient data collection. C.H.N.C. and A.F.D.A.F. performed the statistical analyses. A.F.D.A.F., C.H.N.C., D.L.C., M.S.A.B.S., and K.M.A.V. wrote, revised, and finalised the manuscript.

## Ethics Statement

The study protocol was approved by the Research Ethics Committee of the Federal University of Piauí (CAAE 49960215.3.0000.5214) and Report Number (1.544.071), and informed consent was obtained from all participants or their legal guardians. The project was periodically renewed and monitored to ensure compliance with current ethical guidelines. All procedures followed the principles of the Declaration of Helsinki for research involving human subjects.

## Conflicts of Interest

The authors declare no conflicts of interest.

## Peer Review

The peer review history for this article is available at https://www.webofscience.com/api/gateway/wos/peer‐review/10.1111/pim.70014.

## Data Availability

The authors will provide the raw data supporting the conclusions of this article to any qualified researcher upon reasonable request, without undue restriction.
